# Balancing medicine prices and business sustainability: analyses of pharmacy costs, revenues and profit shed light on retail medicine mark-ups in rural Kyrgyzstan

**DOI:** 10.1186/1472-6963-10-205

**Published:** 2010-07-13

**Authors:** Brenda Waning, Jason Maddix, Lyne Soucy

**Affiliations:** 1 Boston University School of Medicine, Department of Family Medicine; One Boston Medical Center Place, Dowling 5 South, Boston, MA 02118, USA; 2 Utrecht University, Utrecht, Netherlands; 3 Partners in Health, Boston, MA, USA

## Abstract

**Background:**

Numerous not-for-profit pharmacies have been created to improve access to medicines for the poor, but many have failed due to insufficient financial planning and management. These pharmacies are not well described in health services literature despite strong demand from policy makers, implementers, and researchers. Surveys reporting unaffordable medicine prices and high mark-ups have spurred efforts to reduce medicine prices, but price reduction goals are arbitrary in the absence of information on pharmacy costs, revenues, and profit structures. Health services research is needed to develop sustainable and "reasonable" medicine price goals and strategic initiatives to reach them.

**Methods:**

We utilized cost accounting methods on inventory and financial information obtained from a not-for-profit rural pharmacy network in mountainous Kyrgyzstan to quantify costs, revenues, profits and medicine mark-ups during establishment and maintenance periods (October 2004-December 2007).

**Results:**

Twelve pharmacies and one warehouse were established in remote Kyrgyzstan with < US $25,000 due to governmental resource-sharing. The network operated at break-even profit, leaving little room to lower medicine prices and mark-ups. Medicine mark-ups needed for sustainability were greater than originally envisioned by network administration. In 2005, 55%, 35%, and 10% of the network's top 50 products revealed mark-ups of < 50%, 50-99% and > 100%, respectively. Annual mark-ups increased dramatically each year to cover increasing recurrent costs, and by 2007, only 19% and 46% of products revealed mark-ups of < 50% and 50-99%, respectively; while 35% of products revealed mark-ups > 100%. 2007 medicine mark-ups varied substantially across these products, ranging from 32% to 244%. Mark-ups needed to sustain private pharmacies would be even higher in the absence of government subsidies.

**Conclusion:**

Pharmacy networks can be established in hard-to-reach regions with little funding using public-private partnership, resource-sharing models. Medicine prices and mark-ups must be interpreted with consideration for regional costs of business. Mark-ups vary dramatically across medicines. Some mark-ups appear "excessive" but are likely necessary for pharmacy viability. Pharmacy financial data is available in remote settings and can be used towards determination of "reasonable" medicine price goals. Health systems researchers must document the positive and negative financial experiences of pharmacy initiatives to inform future projects and advance access to medicines goals.

## Background

Much of the developing world still lacks access to essential medicines. Most people in developing countries seek care and medicines from private sector pharmacies, even before seeking care at a clinic or hospital [1]. Health systems research must include assessment of pharmacy interventions designed to increase access to medicines given the dominant role pharmacies play in health service delivery.

Access to essential medicines in low resource settings is hindered by high and unaffordable medicine prices [2-7], and global calls to make medicines more affordable have increased in recent years. The Millennium Development Goals include a target that aims "in cooperation with pharmaceutical companies, [to] provide access to affordable essential drugs in developing countries" [8]. The Millennium Development Task Force specifically recommends that countries "seek ways to reduce the trade and distribution mark-ups on prices of essential medicines and to ensure availability of essential medicines in public health care facilities" [8]. The Working Group on Access to Essential Medicines established by the United Nations Millennium Project suggested that generic competition, price negotiation, differential pricing, and effective procurement are the four strongest levers to reduce medicine prices [9].

[10] (delete). The World Health Organization (WHO) and Health Action International (HAI) recently released a methodology to measure medicine price components along the supply chain [11]. The new module is designed to identify where "add-on" prices are applied throughout the supply chain from manufacturer to patient, including duties, taxes, tariffs, and mark-ups [11]. A recent synthesis of WHO/HAI medicine price surveys revealed average retail mark-ups on medicines ranging up to 552% [3], while another summary reported excessive mark-ups specifically in the private sector [11]. These results are compelling and useful for advocates who pressure policy makers to intervene to bring about lower prices. But when confronted with survey results, Ministers of Health inevitably ask: "What is a *reasonable *mark-up for medicines?" This question has yet to be answered. Indeed, the authors of the WHO/HAI synthesis themselves note that additional research is required to determine appropriate medicine mark-ups that are not only reasonable, i.e. as affordable to the consumer as possible, but also ensure the economic viability of the supply chain [3]. This paper is the first publication to respond to this call.

Medicine prices and mark-ups will be difficult to interpret without some basic understanding of the cost, revenue, and profit structures of pharmacy businesses. To remain viable, a pharmacy must be able to recoup its costs and make some minimal profit. While numerous small- and large-scale pharmacies and pharmacy networks have been created to improve access to medicines for the poor [12-32], many have failed due to non-existent or poor quality business plans and financial planning [29]. Health services research, however has failed to provide details of how and why these pharmacy initiatives failed. Organizations and governments will continue to open pharmacies as a means of increasing access to medicines. But, until the financial and managerial success and failures of these initiatives are documented, lessons learned from previous experiences will be lost, the same mistakes will be repeated, and pharmacies will continue to fail.

Health services research is also needed to determine medicine prices that not only advance access goals through affordability but also provide incentives for ownership and management of pharmaceutical enterprises. While affordability is a key determinant of access to medicines, downward pressure on prices to unsustainable levels can actually threaten access by removing incentives for entrepreneurs to own and operate pharmacies, therefore making pharmacies less geographically accessible to consumers. Striking a balance between the availability of medicines and the sustainability of pharmacies is critical, given that the majority of people in developing countries rely on the private sector for essential medicines [1]. This balance becomes even more tenuous in rural regions where population densities are low, pharmacies are scarce or nonexistent, residents have little money, and the few available medicines are expensive. In Kyrgyzstan, more than 80% of the country is covered by mountains and 64% of people live in rural regions [33].

The purpose of this study is to quantify the cost and revenue structures for establishing and maintaining rural pharmacies in Kyrgyzstan and to examine medicine mark-ups to determine if they can be further reduced without jeopardizing the sustainability of these enterprises. In so doing, we provide the first example of how cost accounting methods can be applied to pharmacy financial data to ascertain "reasonable" medicine price and mark-ups. Given the demand for this type of information by researchers [3] and national policy makers, we provide guidance on expanding and replicating this research to advance and support future access to medicines initiatives.

## Methods

For this case study, we applied cost accounting methods to information obtained from a not-for-profit rural pharmacy network to quantify cost, revenue, profit and medicine mark-ups from 2004 to 2007. The retail network is located in Jumgal District of Naryn Province in Kyrgyzstan. Jumgal, like most of Kyrgyzstan, is mountainous, but one of the the most accessible mountainous region to the capital city of Bishkek. The retail network, comprising 12 pharmacies and one warehouse, is managed by a local non-governmental organization (NGO) and operated under a revolving medicine fund mechanism. Established in late 2004 in collaboration with the Kyrgyzstani government, the network was designed specifically to increase access to high quality, affordable medicines in rural villages lacking access to pharmacy outlets. The network is described in detail elsewhere [34].

### Pharmacy network costs

We estimated start-up costs (expenses incurred in establishing the network) and recurrent costs (ongoing costs associated with maintaining the network). We distinguish between fixed recurrent costs - costs that are independent of business volume - and variable recurrent costs - costs that fluctuate depending upon business volume. Examples of fixed recurrent costs include salaries, insurance payments, utilities, and travel from the central office to the region for medicine deliveries. Variable recurrent costs included office supplies, repairs, taxes, nurse-dispenser bonuses, and travel between the warehouse and pharmacies. Cost information was obtained from documents such as purchase receipts, payment invoices, and inventory reports.

While non-product costs to support the central office, warehouse, and each pharmacy could be easily tracked, costs for product purchases were only available for the network as a whole and could not be allocated directly to individual pharmacies.

### Pharmacy network revenues

Pharmacy revenues are limited to income from the sale of products, namely medicines and sundries. Monthly revenue reports for individual pharmacies were provided by the central NGO administration. While inventory information was available on medicine flow through the central office, warehouse, and individual pharmacies, the pharmacies did not keep detailed sales records.

### Pharmacy network profit

Profit is presented for both the network as a whole and for individual pharmacies within it.

Network profit is calculated on a monthly basis as follows:

$$profit_{network}\;=\;revenues_{network}\;-\;costs_{network}$$

Individual pharmacy profit is estimated on a monthly basis as follows:

$$profit_{pharmacy\;i}\;=\;revenue_{pharmacy\;i}\;-\;costs_{pharmacy\;i}$$

where

$$costs_{pharmacy\;i}\;=\;non\text{-}product\;costs_{pharmacy\;i}+\;\frac{non\text{-product}\;costs_{office\;and\;wharehouse}}{12}+\;product\;costs_{network}\;\times \;\frac{revenue_{pharmacy\;i}}{revenues_{network}}$$

This estimation assigns the costs to maintain the central office and warehouse equally across each of the 12 pharmacies. In the absence of pharmacy-specific product purchase and sales records, it also estimates pharmacy-specific monthly product costs as a function of individual pharmacy revenue.

### Medicine mark-ups

The pharmacy network was established in September 2004 and it took a few months to build up sufficient inventory to meet local demand. We, therefore, selected the 50 most profitable products over the 2005-2007 time period. We then calculated mark-ups for these top 50 products in each of the study years (2004, 2005, 2006, 2007). Mark-ups for these products were calculated by ear as follows:

$$retail\;mark\text{-}up\;=\;\frac{retail\;price\;of\;products\;procured\;-\;wholesale\;price\;of\;products\;procured}{wholesale\;price\;of\;products\;procured}$$

Calculations for medicine mark ups utilize warehouse level records for products distributed to pharmacies in a given month.

All cost and revenue estimates are provided in Kyrgyz Som (KGS), although start-up costs are converted to United States (US) dollars to provide value context using a conversion factor of 40 KGS per one US dollar.

## Results

### Pharmacy network start-up costs

**Figure 1:**
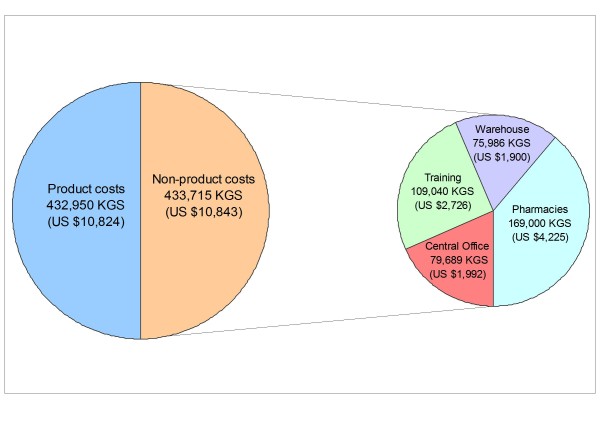
**Start-up costs to establish the pharmacy network**.

The costs to establish the pharmacy network in late 2004 totalled 866,665 KGS (US $21,667), split almost equally across medicine and non-medicine costs (Figure 1). Building pharmacies and training staff accounted for 39% and 25% of non-medicine costs, respectively, while establishing the warehouse and central office each accounted for 18%.

In-kind donations from local Village Health Committees [35] and the Kyrgyz-Swiss Health Reform Support Project also helped establish the network. Such donations included materials and labour to refurbish the pharmacies and warehouse, and some pharmacy furniture (additional file 1).

### Pharmacy network recurrent costs

**Figure 2:**
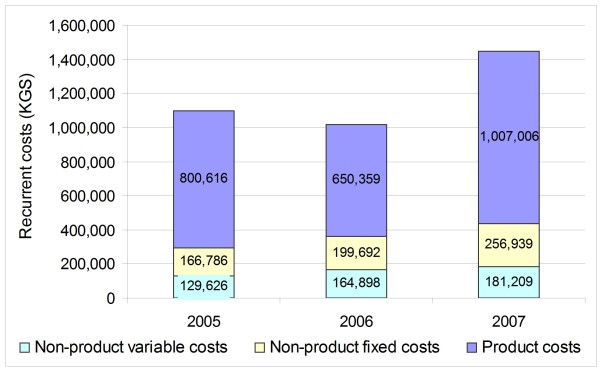
**Overview of recurrent costs (KGS) for pharmacy network**.

Costs for wholesale product purchases vary according to sales volumes in pharmacies. The more products sold by pharmacies each month, the more products the central staff need to purchase from wholesalers to replenish pharmacy stock. As expected, product purchases comprise the largest portion of the variable recurrent costs across all years (Figure 2). All non-product costs increased annually as the newly formed business steadily grew. Non-product variable costs increased 40% from 2005 to 2007, while fixed costs increased 54% from 2005 to 2007, largely due to increased salary expenses for central administrative staff whose contributions were provided in-kind in the first year only. Recurrent cost estimates for 2007 are the most accurate, reflecting the realities of a "mature" network. In 2007, product variable costs, non-product variable costs, and non-product fixed costs comprise 70%, 12%, and 18% of total costs, respectively.

Salaries for central office and warehouse staff represent the greatest portion of non-medicine fixed costs. In 2007, salaries and social insurance payments for employees accounted for 58% and 24% of these costs, respectively (table 1). Travel from Bishkek, the capital city and headquarters, accounted for 17% of fixed costs, and included trips to the warehouse and to the pharmacies for medicine deliveries. The supervisory trips from headquarters were always combined with medicine deliveries to avoid additional travel costs.

**Table 1:** Detailed non-product recurrent costs (KGS) for pharmacy network

	2005	2006	2007
**Non-product fixed costs**			

Central office salaries (supervisors)	44,400	93,600	110,500

Warehouse salaries	39,710	35,835	38,582

Social insurance	45,602	54,557	61,856

Utilities	6,997	2,764	3,413

Travel from central office to region	30,077	12,936	42,588

**Non-product variable costs**			

Office supplies, repair, other	16,230	4,917	15,271

Taxes	4,736	8,159	2,854

Nurse-dispenser bonuses	91,229	125,092	135,694

Travel between warehouse and pharmacies	17,431	26,730	27,390

Nurse-dispenser bonuses, determined by product sales volume, are the largest portion of non-product variable costs, representing 75% of these costs in 2007 (table 1) and averaging 634, 869, and 942 KGS per nurse per month for 2005, 2006, and 2007, respectively. Transport for travel between the warehouse and pharmacies, which includes product deliveries, accounts for 15% of variable costs in 2007.

Cost-sharing arrangements with the Kyrgyzstani Ministry of Health (MOH) allow for very low operating costs. The MOH donated space within primary care clinics to house the pharmacies, on hospital premises to establish the warehouse, and in a Bishkek government building to house the central office. Co-location of the pharmacy network within government facilities means the pharmacy network operates without paying rent or utilities, with the exception of a very modest share of utilities in its central office. In addition, the MOH pays the regular salaries of the nurse-dispensers - who work principally as practicing nurses in the co-located primary care clinics - while the NGO pays the nurses a bonus for taking on the additional task of operating the pharmacies. Whereas the nurses' regular salaries are fixed and subsidized by the Kyrgyz government, the bonuses paid by the NGO are variable, based on pharmacy sales volume.

### Pharmacy network revenues

**Figure 3:**
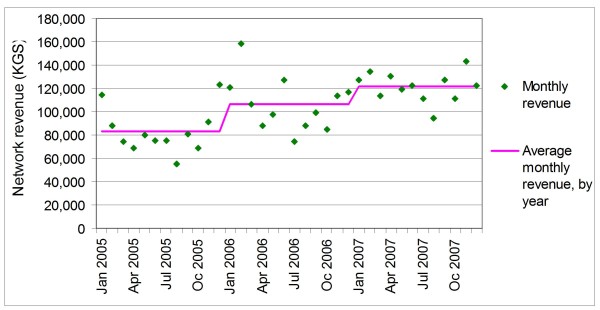
**Monthly revenue and average monthly revenue by year of pharmacy network (KGS)**.

Average monthly revenues increased from 82,837 KGS in 2005 to 121,438 KGS in 2007 (Figure 3). Monthly revenues were highly erratic in the first two years of operation, likely due to inconsistent delivery of stock replenishment to network pharmacies and seasonal variation of medicine use. By, 2007, however, deliveries and revenues had become more stable.

### Pharmacy network profit

**Figure 4:**
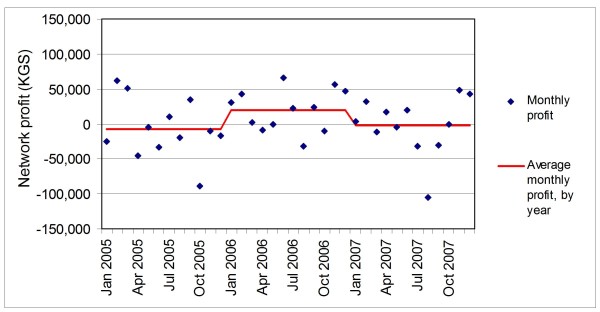
**Pharmacy network monthly profit and average monthly profit by year (KGS)**.

Analyses reveal pharmacy network profits at approximately break-even levels over the entire study period. After operating slightly below break-even levels in 2005, the network averaged small positive profits in 2006 and break-even profit levels in 2007 (Figure 4). Like monthly revenues, monthly expenditures on products and monthly profits were erratic.

### Retail product mark-ups

Mark-ups vary substantially across the network's top 50 products (which account for >50% of network profits), ranging from 32% to 244% in 2007. Those above 150% that might be considered "excessive" cross-subsidize lower mark-ups applied to other medicines. Upward trends in retail mark-ups are noted for nearly all 50 top-selling medicines and health products from 2004 to 2007. Initial mark-ups were low in 2005, the first full year of operation, with 27 (55%) and 17 (35%) products revealing mark-ups of < 50% and 50-99%, respectively (Table 2). Only five (10%) products had a mark-up greater than 100% in 2005. Mark-ups steadily increased from 2005 to 2007 as the NGO was unable to cover its operating costs with the initial mark-ups. By 2007, only nine (19%) and 22 (46%) products revealed mark-ups of < 50% and 50-99%, respectively; while 17 (35%) products showed mark-ups greater than 100%. Mark-up trends for these specific medicines and health products are provided in additional file 2.

**Table 2:** Retail mark-up trends for the 50 top-selling products*, 2004-2007

	# of products per mark-up category			
	**< 50% n (%)**	**50-99% n (%)**	**100-200% n (%)**	**> 200% n (%)**

**2004**	26 (74)	5 (14)	2 (6)	2 (6)

**2005**	27 (55)	17 (35)	3 (6)	2 (4)

**2006**	14 (29)	21 (44)	12 (25)	1 (2)

**2007**	9 (19)	22 (46)	15 (31)	2 (4)

*50 most profitable products 2005-2007. Not all products sold in all years.

## Discussion

This study demonstrates the utility of analyzing financial data obtained from pharmacies to predict costs of establishing new pharmacy businesses and determine reasonable medicine prices and mark-ups that are affordable but still ensure pharmacy viability. Information gained from this type of research can empower policy makers and advocates to develop strategic, evidence-based interventions appropriate for their local context, without jeopardizing sustainability of pharmacy enterprises and availability of medicines.

### Medicine mark-ups needed to ensure non-profit viability were higher than expected and might be considered "excessive" when interpreted without consideration for the cost of business

The level of medicine mark-up needed to sustain the pharmacies was much greater than expected in the planning phase of the project. While the majority of medicines revealed mark-ups of less than 50% upon the initial establishment of the network, mark-ups increased steadily year after year, with few medicines marked-up below 50% and the vast majority marked-up well above 50% and 100% by the end of 2007.

High mark-ups were necessary even given the network's reliance on government subsidies for rent, overhead, and nurses' salaries, as well the in-kind contributions of others. The network's high operating costs for salaries and travel/transport, together with low inventory turnover, translated into high carrying costs for the pharmacy network. This is likely the case in many other rural regions.

### The pharmacy network has few options to lower medicine prices without jeopardizing availability

In order to lower some mark-ups that may be considered "excessive" (e.g. >150%), the management would need to increase mark-ups on other medicines. The NGO could leverage medicine prices and mark-ups in an effort to drive demand of specific products. Mark-ups could be redistributed, applying low mark-ups to encourage the use of key essential medicines and high mark-ups to discourage the use of non essential medicines. Similarly, the NGO should increase mark-ups on sundries (e.g. creams, shampoos, etc.) to maximize revenues and cross-subsidize lower mark ups on medicines. The NGO could also conduct market surveys to identify additional sundries held in high demand by the local community.

While we have not presented the analysis in this paper, we found that profit at the individual pharmacy level varied, with some pharmacies performing better than others [36]. Not surprisingly, pharmacies located in villages with larger populations enjoyed greater profits than those in less-populated villages. Distance from the warehouse in the district center was more closely related to profit, with the most remote pharmacies operating at a slight loss. In this mountainous region, remote pharmacies are located 45-66 kilometers (28-41 miles) from the warehouse. Roads to many of these villages are unpaved and in disrepair, making travel time-consuming and costly, especially during the region's long winters.

It is certainly possible to increase the operational efficiency of these pharmacies, but potential gains would be marginal and insufficient to reduce current medicine prices and mark-ups. Closing pharmacies or reducing the number and type of medicines stocked in villages operating at a loss would increase overall network profit, but at the expense of decreasing access to medicines in the villages most in need. This option would directly contradict the original intention of establishing the network to meet the needs of the least served. Nurse dispenser bonuses should be re-evaluated. Current compensation is based upon sales volume and creates perverse incentives for over-prescribing. These nurse dispenser bonuses account for costs nearly equal to product costs and could be reduced by revising compensation policies.

### Results from analyses of not-for-profit pharmacies can be used to guide policy decisions in for-profit pharmacies

Medicine prices and mark-ups revealed in this study can be used as reliable benchmarks to assess those applied to medicines in for-profit pharmacies in similar regions of Kyrgyzstan. Private sector pharmacies in this region would need to apply even higher retail medicine mark-ups in order to remain profitable in the absence of subsidies. Private pharmacies need to recoup the costs of rent, utilities, and salaries in addition to the costs we included in our analysis of the subsidized, not-for-profit pharmacy network. In addition, the start-up costs in the Kyrgyz pharmacy network were paid up front by others, and therefore, no amortization of these costs was needed; however, private pharmacies would need to amortize these up-front costs over several years. After accounting for these additional costs, advocates can provide policy makers with realistic, evidence-based goals for medicine price determination that ensure the viability and sustainability of pharmacy businesses.

### Medicine markets in rural regions are local, requiring additional localized research with improved methods to better inform decision makers

While these results can be extrapolated to similar rural regions in Kyrgyzstan, they cannot be extrapolated to large cities or more remote regions in Kyrgyzstan or to other low-resource countries. The supply and demand sides of pharmaceutical markets, as well as the business structures of pharmacies, vary dramatically within and across countries. Sound policy decisions can only be made after understanding the unique characteristics of local markets and pharmacy businesses. For example, a WHO/HAI survey in Syria reports a fixed price system whereby retail pharmacies apply maximum medicine mark-ups of 8% for more expensive medicines to 30% for less expensive medicines [37]. If this pricing system was adopted by Kyrgyzstan, the rural pharmacies would fail to thrive and there would be no incentive to open new pharmacies in regions without them. Price controls are often a knee-jerk governmental reaction to high and unaffordable medicine prices; but without understanding local cost of business, imposing arbitrary price and mark-up limitations could jeopardize the availability of medicines and market growth, especially in rural regions.

Our study also revealed the importance of using sampling methods based upon local medicine use patterns. We based our selection of medicines in this study upon sales volume, rather than a pre-determined basket of medicines, to ensure we are measuring prices and mark-ups for medicines that are actually used in the local context. While several hundred items were purchased by the network over the study period, we chose the top selling 50 products because their sales represent more than 50% of all revenues. The top-selling 50 products in terms of profit and volume of sales are similar and represent those products in regular demand while the remaining products are typically purchased only a few times over the entire period. Researchers might consider replicating studies such as ours using volume-based sampling and the local not for profit prices as reference prices.

Research on medicine mark-ups often uses summary measures (such as the average mark-up) across all or a select basket of medicines. Our study found higher mark-ups applied to the more commonly purchased medicines, underscoring the importance of selecting medicines based upon local demand. In addition, we revealed dramatic and unpredictable variation in mark-ups applied across the top selling products, illustrating the limited utility of summary measures and the need to provide detailed results for the entire distribution of medicine mark-ups.

We recognize that it is difficult to obtain financial data on cost of doing business, given the proprietary nature of this information. But we believe this information is available, since most countries have pharmacy networks owned and operated by NGOs or other non profit entities. Typically, these organizations are in the pharmacy business in order to provide quality and affordable medicines to the poor and would likely share their financial information in the interest of national efforts to increase access to medicines. Projects such as the Medicines Transparency Alliance are in a good position to obtain and use this type of information to inform policy, given their multi stakeholder and country-led approaches [38].

### Study limitations

Our study contributes to the growing body of literature on medicine prices, but it has limitations. We had full access to all financial data but expect we missed some unaccounted costs, such as "informal payments" to inspectors, as well as other undocumented revenues. We measure mark-ups at retail level only. The determination of cost of business and mark-up at manufacturer and wholesale level would provide a more comprehensive view of the market, but we had no access to such data.

Because pharmacies did not record medicine-specific sales or information on product losses (e.g., unpaid customer bills, theft, expiration, etc.), we were unable to assess product-specific revenues and costs associated with low turnover. In the absence of pharmacy-specific purchase information, we were unable to measure directly recurrent medicine costs at the pharmacy level. Instead, we estimated these costs as a function of individual pharmacy revenue.

There is no evidence to suggest wholesalers engaged in rebate and bundling practices that are common in developed countries [39,40]. Finally, we present current medicine prices in lieu of adjusting prices for inflation after noting that most medicine prices outside the pharmacy network trended downward or remained unchanged over the four years [34,36] and did not seem to follow the overall national inflation rate of 10% [41].

## Conclusion

Running pharmacy businesses in rural regions is costly, requiring high medicine mark-ups to recoup operating costs and maintain inventory with low turnover. Our study revealed high medicine mark-ups were needed to sustain not-for-profit pharmacies even in the presence of government subsidies and cost-sharing arrangements. Few options to lower medicine prices are available when pharmacies are operating at break-even or low profit levels, but might include interventions to increase operational efficiency; decrease stock levels in low-volume outlets; and redistribute low mark-ups to encourage the use of key essential medicines and high mark-ups to discourage the use of non-essential medicines.

Survey results detailing medicine prices and mark-ups have limited utility without an understanding of regional pharmacy cost, revenue, and profit structures such as those we observed in this study. Policy makers and advocates need this context to set realistic and non-arbitrary goals to reduce medicine prices and mark-ups.

Because medicine prices and mark-ups are locally determined, this type of analysis will need to be replicated in other regions to better inform local and national policies and strategies aimed to increase access to medicines. Interventions must be designed and evaluated to carefully balance medicine prices with pharmacy business sustainability to ensure the availability of medicines in rural regions.

## Competing interests

The authors declare they have no competing interests.

## Authors' contributions

BW designed and coordinated the study, participated in data cleaning and data analysis, and was the lead author on this paper. JM coordinated data collection and management, conducted data analysis, and participated in preparation of the manuscript. LS assisted in data analysis and preparation of the manuscript. All authors read and approved the final manuscript.

## Funding

United States Agency for International Development through the Child and Family Applied Research Project at the Boston University School of Public Health.

## Pre-publication history

The pre-publication history for this paper can be accessed here:

http://www.biomedcentral.com/1472-6963/10/205/prepub

## Supplementary Material

Additional file 1 **Detailed break-down of one-time costs to establish the pharmacy network**. Itemized costs (in USD and Kyrgyz Som) for all products and services needed to establish the pharmacy network. Click here for file

Additional file 2 Trends in average retail mark-ups for 50 top-selling products 2005-2007*. Initial (2004) and average annual percent mark-ups (2005-2007) for the top 50-selling products. *medicines are tablets/capsules unless otherwise noted. ^†^products appear twice representing different pack sizes procured for each product. Click here for file

## References

[B1] World Health Organization World Medicines Situation 2010 Geneva in press

[B2] Babar ZUD Ibrahim MIM Singh H Bukahri NI Creese A Evaluating drug prices, availability, affordability, and price components: implications for access to drugs in Malaysia PLoS Med 2007 4 3 0466 0475 10.1371/journal.pmed.0040082 PMC1831730 17388660

[B3] Cameron A Ewen M Ross-Degnan D Ball D Laing R Medicine prices, availability, and affordability in 36 developing and middle-income countries: a secondary analysis Lancet 2009 373 9659 240 249 10.1016/S0140-6736(08)61762-6 19042012

[B4] Gelders S Ewen M Noguchi N Laing R Price, availability and affordability: An international comparison of chronic disease medicines 2006 Cairo: World Health Organization Regional Office for the Eastern Mediterranean and Health Action International

[B5] Mendis S Fukino K Cameron A Laing R Filipe A Jr Khatib O Leowski J Ewen M The availability and affordability of selected essential medicines for chronic diseases in six low- and middle-income countries Bull World Health Organ 2007 85 279 288 10.2471/BLT.06.033647 17546309 PMC2636320

[B6] Russo G McPake B Medicine prices in urban Mozambique: a public health and economic study of pharmaceutical markets and price determinants in low-income settings Health Policy Plan 2010 25 1 70 84 10.1093/heapol/czp042 19843636

[B7] Coghlan R Auton M M M Reza J, Banerji J Supply chain and price components of antimalarial medicines: Uganda 2007 2008 Geneva: Medicines for Malaria Venture

[B8] Millenium Development Goal Gap Task Force Delivering on the Global Partnership of Achieving the Millenium Development Goals: Millenium Development Goal 8 2008 New York: United Nations

[B9] Leach B Paluzzi JE Munderi P Prescription for healthy development: increasing access to medicines London 2005

[B10] Health Action International Medicine Prices http://www.haiweb.org/medicineprices/

[B11] World Health Organization and Health Action International Measuring medicine prices, availability, affordability and price components 2008 2 Geneva

[B12] Sambo MN Lewis I Sabitu K Essential drugs in primary health centres of north central Nigeria; where is Bamako initiative? Nigerian J Clin Practice 2008 11 1 9 13 18689131

[B13] von Massow F Korte R Cheka C Kuper M Tata H Schmidt-Ehry B Financially independent primary health care drug supply system in Cameroun Trop Med Int Health 1998 3 10 788 801 10.1046/j.1365-3156.1998.00306.x 9809912

[B14] Murakami H Phommasack B Oula R Sinxomphou S Revolving drug funds at front-line health facilities in Vientiane, Lao PDR Health Policy Plan 2001 16 1 98 106 10.1093/heapol/16.1.98 11238436

[B15] McPake B Hanson K Mills A Community financing of health care in Africa: An evaluation of the Bamako initiative Soc Sci Med 1993 36 11 1383 1395 10.1016/0277-9536(93)90381-D 8511627

[B16] United Nations Children's Fund Equity of access in the implementation of the Bamako initiative: research on the Bamako initiative in Vietnam 1994 Hanoi, Vietnam: United Nations Children's Fund

[B17] Hardeman W Van Damme W Van Pelt M Por I Kimvan H Meessen B Access to health care for all? User fees plus a Health Equity Fund in Sotnikum, Cambodia Health Policy Plan 2004 19 1 22 32 10.1093/heapol/czh003 14679282

[B18] Hamada A Essential drug revolving fund programme within the context of pharmaceutical development of Vietnam 1999 Programme: Evaluation of Nippon Foundation Drug Revolving Fund Project in Asia

[B19] Ali GKM How to establish a successful revolving drug fund: the experience of Khartoum state in the Sudan Bull World Health Organ 2009 87 2 139 142 10.2471/BLT.07.048561 19274366 PMC2636186

[B20] Ali GKM Accessibility of medicines and primary health care: The impact of the revolving drug fund in Khartoum State Afr J Pharma Pharmaco 2009 3 3 70 77

[B21] Svhakhang L Sengaloundeth S Freudenthal S Walhstrom R Meessen B, Pei X, Criel B, Bloom G Availability of essential drugs and sustainability of village revolving drug funds in remote areas of Lao PDR Health and Social Protection: Experiences from Cambodia, China and Lao PDF 2008 23 Antwerp: ITGPress 519 543

[B22] Umenai T Narula IS Revolving drug funds: a step towards health security Bull World Health Organ 1999 77 2 167 171 10083717 PMC2557590

[B23] Huff MA Rizal A 20 Years of Revolving Drug Funds: Should the public sector conduct business? Public Health and Human Rights: APHA 134th Annual Meeting and Exposition 2006 Boston, MA

[B24] Fiedler JL Wight JB Financing health care at the local level: the community drug funds of Honduras Int J Health Plan Manage 2000 15 4 319 340 10.1002/hpm.598 11246900

[B25] Diallo I McKeown S Wone I Bamako boost for primary care World Health Forum 1996 17 4 382 385 9060237

[B26] Witter S Achieving sustainability, quality and access: lessons from the world's largest revolving drug fund in Khartoum East Mediterr Health J 2007 13 6 1476 1485 18341197 10.26719/2007.13.6.1476

[B27] Uzochukwu B Onwujekwe O Healthcare reform involving the introduction of user fees and drug revolving funds: influence on health workers' behavior in southeast Nigeria Health Policy 2005 75 1 1 8 10.1016/j.healthpol.2005.01.019 16298224

[B28] Africare Community Health and Partnerships Program Drug Revolving Fund Supervisory Tool: a Job Aid for Supervisors Africare. Washington, D.C 2001

[B29] Cross PN Huff MA Quick JD Bates JA Revolving drug funds: Conducting business in the public sector Soc Sci Med 1986 22 3 335 343 10.1016/0277-9536(86)90132-2 3515574

[B30] Turshen M Reprivatizing pharmaceutical supplies in Africa J Public Health Policy 2001 22 2 198 225 10.2307/3343460 11469153

[B31] Waddington C Panza A Ten questions to ask about revolving drug funds Trop Doct 1991 21 2 50 53 1871877 10.1177/004947559102100202

[B32] Gilson L Kalyalya D Kuchler F Lake S Oranga H Ouendo M Strategies for promoting equity: experience with community financing in three African countries Health Policy 2001 58 1 37 67 10.1016/S0168-8510(01)00153-1 11518601

[B33] Central Intelligence Agency World Fact Book 2009 Central Intelligence Agency

[B34] Waning B Maddix J Tripodis Y Laing R Leufkens H Gokhale M Towards equitable access to medicines for the rural poor: analyses of insurance claims reveal rural pharmacy initiative triggers price competition in Kyrgyzstan Int J Equity Health 2009 8 1 43 10.1186/1475-9276-8-43 20003422 PMC2803474

[B35] Formation of Village Health Committees http://cah.kg/en/formation_of_village_health_committees/

[B36] Waning B Maddix J Djankorozova M Gokhale M Winter M Diedrichsen E Jafarov A Improving rural access to medicines in Kyrgyzstan: a view through the lenses of insurance, households, communities, and retail pharmacy business 2009 Boston: Boston University School of Medicine

[B37] World Health Organization and Health Action International Syrian Arab Republic: medicine prices, availability, affordability, and price components 2003 Cairo: World Health Organization Regional Office for the Eastern Mediterranean

[B38] Medicines Transparency Alliance http://www.medicinestransparency.org/

[B39] Kalmanowitz S Drug/technology bundling: Good for you? Drug Topics 2004 148 24

[B40] Frank RG Prescription Drug Prices: Why Do Some Pay More Than Others Do? Health Aff 2001 20 2 115 128 10.1377/hlthaff.20.2.115 11260933

[B41] International Monetary Fund World Economic Outlook Database Washington DC 2009

