# Supplementary material for: Balancing medicine prices and business sustainability: analyses of pharmacy costs, revenues and profit shed light on retail medicine mark-ups in rural Kyrgyzstan
Source: BMC Health Serv Res. 2010 Jul 13;10:205. doi: 10.1186/1472-6963-10-205 (PMC2914726; doi:10.1186/1472-6963-10-205)
Supplement: Additional file 1 — Detailed break-down of one-time costs to establish the pharmacy network. Itemized costs (in USD and Kyrgyz Som) for all products and services needed to establish the pharmacy network. [file 1472-6963-10-205-S1.DOC]

Additional file 1. Detailed break-down of one-time costs to establish the pharmacy network

| **One-Time Costs** | **Kyrgyz**  **Som** | **US**  **Dollars** |
| --- | --- | --- |
| **Product costs**  **(medicines & sundries)** | **432,950** | **10,824** |
| **Non-product costs** | **433,715** | **10,843** |
| *Central administrative office* | *79,689* | *1,992* |
| License and certificate fees | 2,945 | 74 |
| Banking fees | 3,768 | 94 |
| Informational materials | 13,579 | 339 |
| Official stamp | 1,170 | 29 |
| Office supplies | 10,487 | 262 |
| Travel to warehouse & pharmacies | 47,740 | 1,194 |
| *Training* | *109,040* | *2,726* |
| *Warehouse* | *75,986* | *1,900* |
| Computer, printer, surge-protector | 34,002 | 850 |
| Telephone and fax machine | 10,297 | 257 |
| Furniture (desk, chair, shelving) | 20,000 | 500 |
| Metal bars for security (installed) | 6,550 | 164 |
| Renovation | 3,137 | 78 |
| Safe | In-kind | In-kind |
| Other | 2,000 | 50 |
| *Pharmacies* | *169,000* | *4,225* |
| Display safe and shelving | 78,000 | 1,950 |
| Locked cabinet and safe | In-kind | In-kind |
| Chair | In-kind | In-kind |
| Window shades | 6,500 | 163 |
| Metal bars for security (installed) | In-kind | In-kind |
| Signs | 84,500 | 2,113 |
| Renovation | In-kind | In-kind |
| Calculator | In-kind | In-kind |
| **Total** | **866,665** | **21,667** |
