# Supplementary material for: Balancing medicine prices and business sustainability: analyses of pharmacy costs, revenues and profit shed light on retail medicine mark-ups in rural Kyrgyzstan
Source: BMC Health Serv Res. 2010 Jul 13;10:205. doi: 10.1186/1472-6963-10-205 (PMC2914726; doi:10.1186/1472-6963-10-205)
Supplement: Additional file 2 — Trends in average retail mark-ups for 50 top-selling products 2005-2007*. Initial (2004) and average annual percent mark-ups (2005-2007) for the top 50-selling products. *medicines are tablets/capsules unless otherwise noted. †products appear twice representing different pack sizes procured for each product. [file 1472-6963-10-205-S2.DOC]

Additional file 2. Trends in average retail mark-ups for 50 top-selling products*

| **Sales**  **Rank** | **Product** | **Initial Mark-up Oct-Dec 2004** | **Average**  **Mark-Up**  **2005** | **Average**  **Mark-Up**  **2006** | **Average**  **Mark-Up**  **2007** |
| --- | --- | --- | --- | --- | --- |
| 1 | amoxicillin 250mg^†^ | - | 92% | 102% | 109% |
| 2 | Bicillin-5® 1500IU injection  (penicillin G) | 36% | 39% | 49% | 85% |
| 3 | benzylpenicillin injection | 275% | 43% | 43% | 43% |
| 4 | ampicillin 500mg injection | 29% | 25% | 32% | 33% |
| 5 | ciprofloxacin 250mg | - | 83% | 129% | 164% |
| 6 | disposable syringe 5ml | 77% | 87% | 91% | 88% |
| 7 | ampicillin 250mg^†^ | 46% | 99% | 104% | 114% |
| 8 | paracetamol 500mg | 71% | 130% | 111% | 112% |
| 9 | Synaflan® 0.025% ointment  (fluocinolone) | 30% | 125% | 99% | - |
| 10 | salbutamol inhaler | 27% | 32% | 39% | 93% |
| 11 | Antigrippin® | 39% | 35% | 45% | 65% |
| 12 | ascorbic acid 100mg | 48% | 63% | 121% | 140% |
| 13 | acetylsalicylic acid 500mg | 132% | 141% | 101% | 113% |
| 14 | paracetamol 200mg | 219% | 233% | 231% | 242% |
| 15 | erythromycin 250mg^†^ | - | 36% | 54% | 69% |
| 16 | Ednyt 20mg®  (enalapril) | - | 54% | 45% | 53% |
| 17 | atenolol 50mg | 32% | 39% | 111% | 140% |
| 18 | Antigrippin P® | 46% | 41% | 39% | 69% |
| 19 | Levomycetin® 500mg  (chloramphenicol) | 25% | 47% | 69% | 67% |
| 20 | Novocaine® 0.5% injection; 5ml (procaine)^†^ | 30% | 45% | 50% | 34% |
| 21 | Ferrum Lek® 100mg  (iron hydroxide) | - | 30% | 39% | 43% |
| 22 | Biseptol® 480mg  (cotrimoxazole) | - | 44% | 50% | 50% |
| 23 | carbamazepine 200mg | - | 41% | 76% | 82% |
| 24 | Bicillin-3® 600 IU  (penicillin G) | 59% | 70% | 60% | 66% |
| 25 | Teturam® 0,15 | 41% | 59% | 61% | 69% |
| 26 | Tardyferon® 80mg  (ferrous sulphate) | 16% | 10% | 13% | 19% |
| 27 | Novocaine® 0.5% injection; 5ml (procaine)^†^ | - | 232% | 51% | 69% |
| 28 | ampicillin trihydrate 250mg^†^ | - | 43% | - | 119% |
| 29 | Citramon-P®  (combination analgesic) | 115% | 98% | 113% | 103% |
| 30 | Linkas syringe 90ml | - | 37% | 37% | 41% |
| 31 | Pregnancy test | 41% | 66% | 131% | 156% |
| 32 | metronidazole 250mg | 41% | 41% | 97% | 112% |
| 33 | cinnarizine 25mg | 43% | 47% | 96% | 112% |
| 34 | Ednyt® 10mg (enalapril) | - | 56% | 45% | 60% |
| 35 | cotrimaxazole 480mg | 58% | 93% | 74% | 176% |
| 36 | erythromycin 250mg^†^ | 22% | 45% | 50% | 48% |
| 37 | tetracycline 100mg | - | 86% | 110% | 56% |
| 38 | gentamicin 80mg injection | 30% | 44% | 59% | 60% |
| 39 | Metrid 0,5% 100,0 | 34% | 37% | 59% | 98% |
| 40 | atenolol 100mg | 33% | 39% | 111% | 140% |
| 41 | cotton wool 25gm | 35% | 22% | 121% | 244% |
| 42 | metronidazole 500mg suppositories | 33% | 61% | 89% | 84% |
| 43 | Shampoo | - | - | - | 96% |
| 44 | Analgin® 500mg | 50% | 68% | 62% | 70% |
| 45 | diclofenac injection | 35% | 26% | 49% | 69% |
| 46 | syntomycin 10% ointment | 37% | 57% | 88% | 105% |
| 47 | amoxicillin 250mg^†^ | 39% | 42% | 84% |  |
| 48 | Lodomarin® 0.2mg  (potassium iodide) | - | 33% | 39% | 28% |
| 49 | Soap | - | 46% | 27% | 32% |
| 50 | Disposable Syringe 10ml | 57% | 54% | 56% | 66% |

*medicines are tablets/capsules unless otherwise noted

^†^products appear twice representing different pack sizes procured for each product
